# Xylitol: A Promising Sweetener Produced by *Candida* sp.

**DOI:** 10.1007/s12088-025-01456-1

**Published:** 2025-03-15

**Authors:** Diana Carolina Tusso Pinzón, Margareth Andrea Patiño Lagos, Ricardo Andrés Tusso Pinzón, Liseth Suárez Osorio, Andrés Mauricio Pinzón Velasco, Mario Enrique Velásquez Lozano

**Affiliations:** 1 Facultad de Ciencias, Instituto de Biotecnología, Universidad Nacional de Colombia, Bogotá D.C, Colombia; 2 Facultad de Ingeniería, Departamento de Ingeniería Química y Ambiental, Grupo de Investigación en Procesos Químicos y Bioquímicos, Universidad Nacional de Colombia, Bogotá D.C, Colombia; 3 Facultad de Ciencias Básicas, Grupo de Investigación GIEMA, Universidad Santiago de Cali, Cali, Colombia; 4 Instituto de Genética, Laboratorio de Bioinformática y Biología de Sistemas, Universidad Nacional de Colombia, Bogotá D.C, Colombia

**Keywords:** Xylitol, Yeasts, Biotechnology, *Candida* sp., *Candida tropicalis*

## Abstract

Xylitol is a sugar alcohol that has low-glycemic sweetener suitable for diabetics. It prevents cavities, functions as a prebiotic, and has emollient and moisturizing properties. Xylitol uses as a food additive, sweetener, and dental products. Although the global xylitol market is growing, driven mainly by the chewing gum industry, its production depends on expensive and unsustainable chemical methods, which has motivated the development of biotechnological alternatives. This review examines biotechnological xylitol production in *Candida* species. These yeasts convert efficiently xylose-containing biomass into xylitol, presenting a more economical and eco-friendly alternative to traditional extraction methods. The review will discuss xylitol applications, microbial production, advantages, production parameters, specific strains used, advantages and challenges.

**Supplementary Information:**

The online version contains supplementary material available at 10.1007/s12088-025-01456-1.

## Introduction

Xylitol (C_5_H_12_O_5_) has five hydroxyl groups attached to carbon atoms. Equally sweet as sucrose but with fewer calories (2.4 vs. 4 cal/g) and a lower glycemic index, it is suitable for low-calorie products and diabetic diets, as it is absorbed without insulin, preventing blood sugar spikes [1]. It reduces dental caries by 85% in adults since cariogenic bacteria cannot metabolize it, making it an ingredient in toothpastes and mouthwashes [1]. Xylitol nasal solutions can prevent and treat conditions like osteoporosis, haemolytic anaemia, acute otitis media, sinusitis, and respiratory inflammation [2]. Xylitol is also used to produce compounds like xylaric acid, glycols, hydroxyfuran, polyester resins, PET bottling, paints, and coatings, and is considered a potential low-cost petrochemical substitute with positive market implications [1, 3].

The global xylitol market, valued at $823.6 million in 2018, is projected to reach $1.37 billion by 2025, driven primarily by the chewing gum industry. Suppliers such as Dupont, Mitsubishi Shoji, and Cargill dominate the market [4, 5]. While current production relies on chemical processes, these methods have drawbacks that increase costs and harm the environment. As a result, researchers have explored alternative approaches to address these limitations [6].

Biotechnological methods using yeasts are preferred over chemical methods due to several advantages: no need for xylose purification and crystallization, absence of high pressure and temperature requirements, and the use of certain hemicellulose hydrolysate impurities as nutrients, reducing production costs. Companies like Thomson Biotech, ZuChem Inc., Danisco, and Xyrofin are piloting biotechnological xylitol production, not yet industrialized [1].

Yeasts play an essential role in the biosynthesis of biotechnologically significant chemical compounds, including alcohols, fatty acids, and esters. Among these, *Saccharomyces cerevisiae* is one of the most extensively studied eukaryotic microorganisms, recognized as an exemplary model organism in aging research [7]. However, this yeast lacks the ability to metabolize xylose. In contrast, yeasts of the genus *Candida *sp. are widely employed in xylitol production due to their highly efficient conversion of xylose into xylitol, their remarkable tolerance to acidic conditions and elevated temperatures, and their superior production yields, which can exceed 80% in certain strains. This review explores the critical aspects of xylitol production utilizing *Candida *sp. yeasts, focusing on production methodologies, optimization strategies, and the associated benefits and challenges in harnessing these yeasts for xylitol biosynthesis.

## Xylitol Production

Xylitol is industrially synthesized through the chemical reduction of xylose under high pressures and temperatures using a metal catalyst, typically Raney nickel [8]. The process begins with the hydrolysis of lignocellulosic biomass, breaking down hemicellulose into monomeric sugars to produce a xylose-rich hydrolysate [9]. The hydrolysate undergoes purification to eliminate unwanted byproducts and residues from hydrolysis, with activated carbon and ion exchange chromatography being the most effective methods for decolorization and inhibitor removal [10].

Xylose hydrogenation is carried out at temperatures between 353 and 413 K and hydrogen pressures up to 5 MPa, followed by steps involving purification, concentration, and crystallization to obtain xylitol [11]. The catalyst is separated through filtration and ion exchange, while the crystallization process—utilizing cooling, evaporation, or precipitation—achieves yields exceeding 75% and purities over 98% [9, 10]. Despite these high yields, the chemical production of xylitol is hindered by drawbacks such as high energy demands, the necessity of elevated pressures, and the sensitivity of the catalyst, all of which contribute to increased production costs [6].

## Xylitol Production by Yeasts

**Fig. 1:**
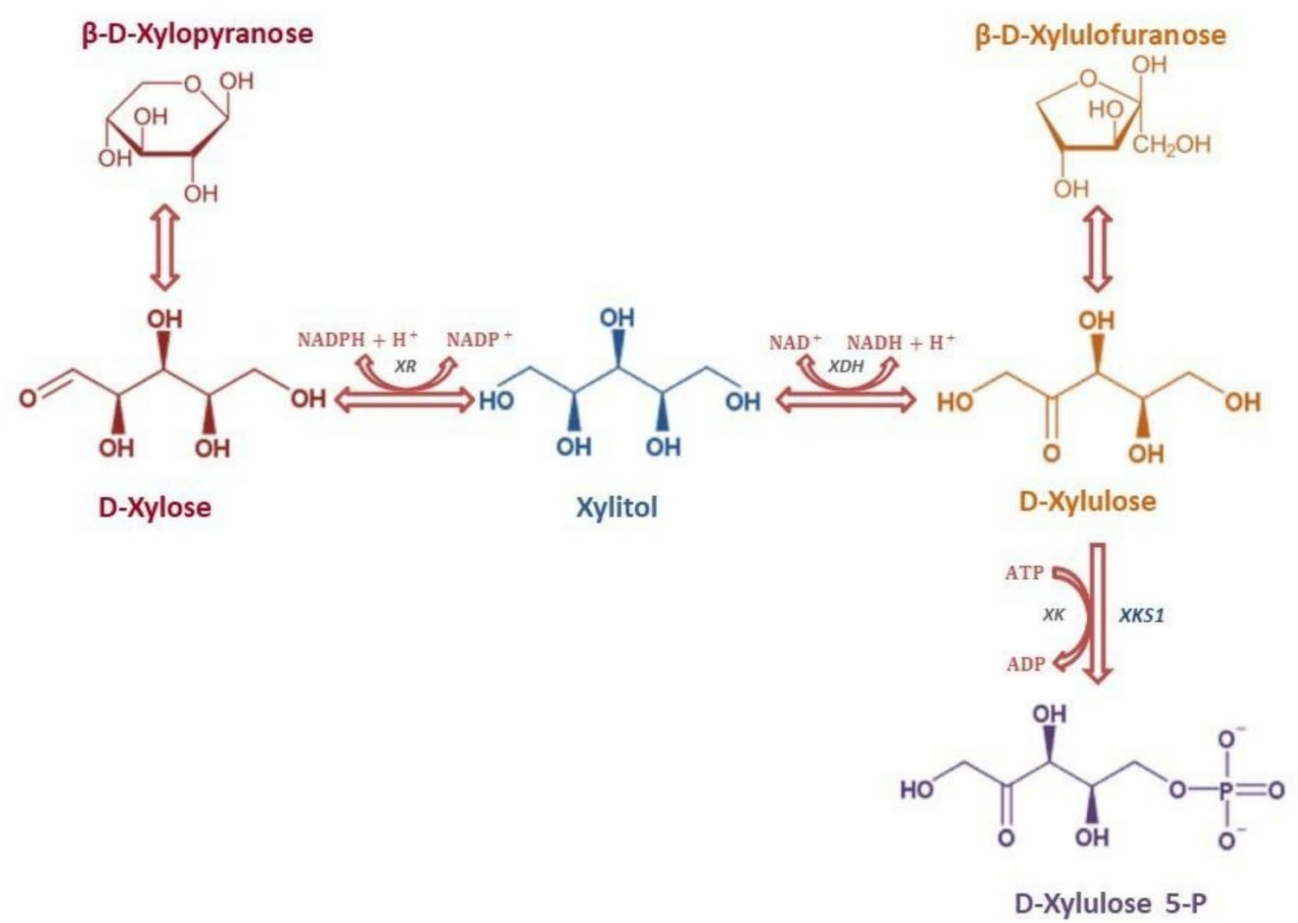
Xylitol biosynthesis in yeast [13]

Xylitol is produced by bacteria, filamentous fungi, and yeasts, with most research focusing on *Candida* species [12]. In yeast, xylose reductase (XR) is the key enzyme for converting hemicellulosic sugars to xylitol, reducing D-xylose to xylitol. Xylitol dehydrogenase (XDH) then oxidizes xylitol to D-xylulose, which is converted to xylulose 5-phosphate by xylulose kinase and enters the pentose phosphate pathway. XDH requires NAD^+^ as a cofactor, while XR uses NAD(P)H, making the efficiency of xylose assimilation dependent on XR and XDH activities (Fig. 1) [13].

Xylitol accumulates under low oxygen conditions due to a redox imbalance of NADH/NAD^+^, which reduces XDH activity because of NAD^+^ shortage. This imbalance occurs as oxidative phosphorylation cannot reoxidize all NADH, enhancing XR activity and xylitol accumulation [9]. In *C. tropicalis*, XR depends on both NADPH and NADH, whereas in *Candida guilliermondii*, it is strictly NADPH-dependent. Consequently, *C. guilliermondii* accumulates acetate to regenerate NADPH, whereas *Candida tropicalis* uses fructose-6-phosphate in the pentose phosphate pathway [13–15]. For NAD^+^ regeneration, both species produce by-products like glycerol and ethanol in low concentrations, serving as a redox drain to maintain XDH activity.

The theoretical maximum yield under aerobic conditions is unaffected by cofactor type, with 0.9 mol xylitol produced/mol xylose consumed using NADPH^2+^ and 0.905 mol/mol with NADH^2+^. The overall aerobic reaction with NADH^2+^ is (Eq. 1.1) [16]:

1.1 $$126\, xylose + 3\, O_{2} + 6 \,ADP + 6\, P_{i} + 48\, H_{2} O \to 114\, xylitol + 6\, ATP + 60 \,CO_{2}$$

Under anaerobic conditions, the theoretical maximum yield is 0.875 mol xylitol produced/mol xylose consumed, as shown in Eq. 1.2 [16].

1.2 $$48\, xylose + 15 \,H_{2} O \to 42\, xylitol + 24\, CO_{2} + 3\, ethanol$$

## Factors Influencing Xylitol Production in *Candida* sp.

Oxygen supply is critical for xylitol production, with its effects studied in synthetic media and hemicellulosic hydrolysates, though varying measurement methods complicate result comparison [9]. Microaerophilic conditions optimize production by regenerating NAD + and generating ATP and NADPH needed for cell growth and XR activity [17]. Initial xylose concentration greatly impacts production, with low levels favoring biomass over yields, while excessive levels lower yield due to osmotic pressure [5, 18, 19]. The carbon-to-nitrogen ratio and nitrogen sources also influence production. Lower ratios promote biomass, while higher ratios enhance metabolite synthesis [20]. Temperature is another key factor, with mesophilic yeasts performing best. Temperatures above 45 °C reduce yields, likely due to XR cofactor inactivation [6]. Yeasts typically grow at pH 4–6, with *Candida parapsilosis* and *C. guilliermondii* thriving at pH 6 and *C. tropicalis* tolerating pH 2.5–4.5 [21, 22].

Microorganism activity and xylitol production are influenced by controlled culture conditions. Optimization strategies for yield, productivity, and product concentration include culture medium design, fermentation process adjustments, and genetic modification, as detailed in the following sections.

### Batch Cultivation

In batch culture, all components of the culture medium and inoculum are added only at the beginning of the fermentation assay, with intra- or extracellular products collected at the end. Conditions like pH, temperature, and dissolved oxygen are typically controlled and remain constant throughout the assay [23].

Table 1 lists studies on xylitol production using batch cultures of *Candida* species, with *C. tropicalis* reported substrate yield (Y_P/S_) ranges from 0.53 to 0.93 g/g, and xylitol final concentrations span 49 to 172 g/L. Notably, *C. athensensis* achieved 207.8 g/L xylitol with an initial xylose concentration of 250 g/L in a yeast extract medium [23]. Regarding the volumetric productivities of the product g/Lh (Q_P_), the values vary between 0.2 and 3.86.

**Table 1:** Studies reported for xylitol production by batches in *Candida* sp.

Strain	Xylose (g/L)	Time (h)	Xylitol (g/L)	Y_P/S_ (g/g)	Q_P_ (g/Lh)	Annotation	References
*Candida tropicalis* IFO 0618	150	42	84.5	0.56	2.01	Glass column reactor 250 mL	[60]
		32	93.9	0.63	2.94	Reactor 3L	
*Candida tropicalis* BSXDH-3	50	16	48.6	0.98	3.23	Semi-defined, with glycerol and glucose Yeast with silencing of the XDH gene	[38]
*Candida athensensis* SB18	250	180	207.8	0.83	1.15	Yeast extract	[23]
*Candida tropicalis* ATCC 20311	200	NR	130	0.93	NR	Semi-defined	[61]
*Candida tropicalis* KCTC 10457	200	47	166	0.83	3.53	Semi-defined	[28]
		47	172	0.86	3.66	With glucose Semi-defined	
*Candida tropicalis* ATCC 13803	100	NR	86	0.8	3.86	Glucose as co-substrate in complex media	[26]
*Candida guilliermondii* FTI20037	60	132	36.56	0.648	0.277	Reactor 2.4 L	[62]
		132	38.28	0.665	0.290	Reactor 18 L	
	40	60	18.6	0.55	0.31	Reactor 125 L	
*Candida tropicalis* KCTC 7221 (ATCC 13803)	150	45	116	0.78	2.6	Complex medium	[29]
		48	109	0.74	2.3	Medium defined	
*Candida parapsilosis* KFCC 10875	50	11	34	0.68	3.09	Yeast mutant of *C. parapsilosis* ATCC 22019 Semi-defined	[63]
	90	20	63	0.70	3.15		
	140	30	103	0.74	3.43		
	170	35	125	0.74	3.57		
	220	52	159	0.72	3.06		
	270	85	189	0.70	2.22		
*Candida boidinii* NRRL Y-17213	130	240	57.5	0.45	0.24	Semi-defined	[64]
*Candida guilliermondii* IM/UFRJ	109	100	NR	0.71	0.85	Semi-defined	[65]
*Candida tropicalis* YHJ1	100	142	66.9	0.67	NR	Medium defined	[66]
*Candida tropicalis* A26	80	42	71.59	0.89	NR	Complex medium	[67]
*Candida guilliermondii* FTI 20037	65	70	39.1	0.6	0.55	Medium with rice bran	[68]
*Candida tropicalis* W103	120	64	87.6	0.73	1.37	Semi-defined	[69]
*Candida tropicalis* As 2.1776	80	96	58.3	0.74	0.61	Corn cob hydrolyzate	[70]
*Candida tropicalis* MTCC 6192	45	96	25.8	0.60	0.26	Rice straw hydrolysate	[71]
*Candida tropicalis* ATCC 13803	50	48	41	0.83	1.87	With glycerol	[72]
*Candida tropicalis* K2	72	100	79	0.78	1.47	Pure Xylose	[73]
*Candida tropicalis* 13,803	100	100	65.15	0.66	1.2	Pistachio shell hydrolysate	[74]
*Candida tropicalis* AY2007	45.5	71	30.99	0.701	0.428	Semi-defined	[75]
		65	36	0.704	0.506		
	96.9	78	14.05	0.18	0.134		
	29.87	94	22.56	0.783	0.239	Hydrolyzed sugarcane bagasse	

NR No information is reported

### Fed-batch Cultivation

In the fed-batch process, nutrients essential for growth and product formation are continuously added, and the product is recovered at the end. This process can be repeated multiple times as long as the cells remain viable and productive [24].

Table 2 illustrates studies on xylitol production by fed-batch in *Candida* species. Variations in carbon source feeding methods were observed, including pulse feeding and constant flow to maintain initial concentrations; both xylose and glucose were used to optimize xylitol production. Yields and productivity improved compared to batch experiments, with Y_P/S_ values of 0.68 (g/g) and Q_P_ of 0.97 (g/Lh).

**Table 2:** Studies reported for xylitol production by fed batch in *Candida* sp.

Strain	Time (h)	Xylitol (g/L)	Y_P/S_ (g/g)	Q_P_ (g/Lh)	Annotation	References
*Candida tropicalis* KCTC 7221 (ATCC 13803)	108	229	0.85	2.1	Yeast extract	[76]
	140	233	0.89	1.7	Urea and vitamins	
	120	237	0.89	2	Vitamins	
*Candida tropicalis* KFCC-10960	68	246	0.82	3.62	Without glucose	[77]
	55	251	0.93	4.56	Added glucose	
*Candida tropicalis* As 2.1776	120	96.5	0.83	1.01	Corn cob hydrolyzate	[70]
*Candida tropicalis* IFO 0618	42	102.2	0.68	2.43	Glass column reactor 250 mL Glucose feeding	[60]
	32	104.5	0.82	3.26	Reactor 3L Glucose feeding	
*Candida tropicalis* ATCC 13803	48	187	0.75	3.9	Complex medium	[18]
*Candida parapsilosis* KFCC 10875	66	210	0.70	3.18	Semi-defined	[35]
*Candida tropicalis* KCTC 10457	57	224	0.86	3.93	Without glucose Semi-defined	[28]
	48	234	0.90	4.88	With glucose Semi-defined	
*Candida tropicalis* SS2	67	220	0.93	3.3	Strain mutant	[78]
*Candida athensensis* SB18	264	256.5	0.87	0.97	Yeast extract	[23]
*Candida tropicalis* W103	120	218.7	0.73	1.82	Semi-defined	[69]

### Other Cultivation Methods

In addition to batch and fed-batch cultures, continuous [25] and recirculation culture [26] have been studied. Continuous culture maintains high productivity over extended periods, but faces issues like mutation induction, cell contamination and accumulation in system tubes [27]. While centrifugation can enhance cell concentration and yield, its large-scale application is costly and contamination-prone [28, 29]. Conversely, Kwon and collaborators developed a cell recirculation system based on a submerged membrane bioreactor with suction pressure and air spray [28].

Xylitol production has also been evaluated using immobilized yeasts; using various bioreactors such as, stirred tank [19, 30–32], bubble column [33], and packed bed reactors [34], showed no significant productivity gains due to limited oxygen transfer, reducing overall efficiency [27].

## Genetic Engineering in Xylitol Production

Despite high yields and productivity in fermentations, native *Candida* sp. yeasts face limitations for industrial-scale xylitol production. High product concentrations, initial substrate levels, resistance to inhibitors produced by hydrolysis of lignocellulosic biomass and by-product formation can inhibit growth and productivity, complicating purification and separation.

Enhancing XR enzyme activity can be achieved through overexpression of a native enzyme or heterologous insertion. Overexpression of the codon-modified XR gene from *Neurospora crassa* in a *C. tropicalis* mutant strain using a constitutive glyceraldehyde 3-phosphate dehydrogenase promoter increased 35 and 73% xylitol productivity [35, 36]. Insertion of the *XYL1* gene from *C. parapsilosis* KFCC-10875 into *C. tropicalis* showed no differences in xylitol, ethanol, and glycerol production compared to the parental strain [37].

To reduce XDH activity and prevent xylitol oxidation, the *XYL2* gene encoding XDH in *C. tropicalis* was deleted. The mutant strain required glycerol as a co-substrate to grow in mineral medium with xylose as the sole carbon source. The *C. tropicalis ΔXYL2* strain converted xylose to xylitol with a productivity of 3.23 g/Lh and a yield of 0.98, accumulating xylulose [38]. The *XYL2* gene has been modified by silencing one copy and introducing point mutations in the cofactor binding region to reduce XDH activity. This increased the Y_P/S_ yield from 0.62 to 0.75, though the mutant grew more slowly [39]. Additionally, enhancing XR activity by increasing NADPH availability was pursued, overexpressing *ZWF* and *GND* genes, encoding glucose-6-phosphate dehydrogenase and 6-phosphogluconate dehydrogenase, was conducted in *C. tropicalis* BSXDH-3, lacking the *XDH* gene, driven by the constitutive glyceraldehyde 3-phosphate dehydrogenase promoter, resulted in a 21% productivity increase compared to BSXDH-3, using glycerol as a co-substrate [40].

Even though improvements in yields and productivity in xylitol production have been observed, challenges remain in developing efficient genetic engineering techniques and overcoming metabolic bottlenecks. The issue of by-product formation persists, hindering the purification of the final product, along with the presence of co-substrates in the culture medium, which increases production costs.

Random mutagenesis and targeted modifications played a key role in developing *C. tropicalis* strains with reduced XDH activity. The EMS-mutated *C. tropicalis* K2M strain increased xylitol production by 9.2%, while the heterozygous *XYL2* strain K21D showed a 16% improvement compared to the parental strain, eliminating the need for co-substrates in xylitol-ethanol biorefinery processes. The genetic modification involved altering one allele of the *XYL2* gene [41](*XYL2δ::FRT*), reducing xylitol dehydrogenase activity and increasing xylitol production.

Recent studies shifted from using pure xylose to renewable sources like xylose mother liquor (XML), a by-product of xylose purification that contains toxic compounds from lignocellulosic biomass hydrolysis, hindering xylitol production. In *C. tropicalis* XZX-B4ZG, *XYL2* genes encoding XDH were deleted to block xylitol conversion into xylulose. An NADPH regeneration system was introduced by expressing *YlZWF*, *YlGND*, and *YlMAE* genes from *Y. lipolytica*. The modified strain produced 97.10 g/L of xylitol in 120 h from 300 g/L XML in a 5-L fermenter, with a 92.40% conversion rate, showing high tolerance to XML inhibitors. However, glucose was used as a co-substrate to maintain intracellular NADPH levels, enhancing XR enzyme activity [42].

Regarding more recent techniques such as CRISPR/Cas9 genome editing for simultaneous deletion-insertion strategies in strain development, these have not been directly applied to strains of the *Candida* species. Instead, genes encoding the XR enzyme from *C. tropicalis* have been overexpressed in *S. cerevisiae*. As a result, xylitol productivity and yield of 0.83 g/Lh and 0.99 g/g, respectively, were achieved using glucose as a co-substrate [43]. Additionally, this tool has been developed for *Candida glycerinogenes*, but its application has mainly focused on ethanol and xylonic acid production [44].

This highlights a research gap in the application of this emerging technique to non-conventional yeasts such as those from the genus *Candida*. One reason is that more standardized protocols exist for model organisms like *S. cerevisiae*, while they are less developed for *Candida*. Another challenge is that some species in this genus have diploid or even polyploid genomes, which can complicate genetic editing. Nevertheless, with the continuous development of genetic engineering tools, it is likely that this technology will eventually be adopted for xylitol production in yeasts of the *Candida* genus.

## Kinetic Models and System Biology for Xylitol Production

A key method for studying xylitol production in *Candida* yeasts uses mathematical models to evaluate fermentation parameters. Kinetic models predict key factors like growth rate, yields, concentrations, and maintenance coefficients, and help analyze fermentation stability [45].

The first kinetic model for xylitol production in *Candida* sp. used an unstructured approach to analyze batch cultures with two stages: in stage A, the microorganism reaches µ_max_ due to excess substrates, while in stage C, oxygen transfer limits growth. Xylitol is consumed without accumulation in stage A and produced in association with growth in stage C. Experimental data across aeration conditions (0.1–0.5 vvm) showed the model accurately represents the variables, with the highest Y_P/S_ value at 0.1 vvm, as detailed in Table S1 [46].

Conversely, Monod, Contois, and Tessier models evaluated growth and substrate consumption in *C. guilliermondii*. Incorporating µ_max_ and saturation constants, they determined cell mass formation and substrate use. The Contois model, with the lowest standard deviation, proved most reliable under the tested conditions [17].

Kim et al. developed a model to describe growth and xylose conversion in *C. tropicalis* 13,803, optimizing initial glucose concentration. The Luong equation, relating ethanol concentration to growth rate, was used to fit batch culture data. Model validation showed a xylitol productivity of 5.06 g/Lh, closely matching predictions [47].

Also, a model predicting biomass, glucose, xylose, and xylitol concentrations in *C. mogii* with 11 parameters (Table S1) was validated with different batch culture assays. While it aligned with xylose consumption trends, post-10-h culture and xylitol concentrations were higher than experimental data [48].

Otherwise, an unstructured dynamic kinetic model for xylitol production and growth in *C. tropicalis* uses kinetic parameters based on initial xylose and oxygen concentrations related to agitation. Although the fermentation profiles from experimental and model predictions showed similar behaviors, errors exceeded 15%, except at 100 g/L xylose, where errors ranged from 4 to 10%, with a 17% in xylitol concentration [49].

In recent years, a co-substrate model for xylitol production was developed using *C. tropicalis* TISTR 5306, incorporating a modified Monod equation to account for substrate limitations and inhibitions, achieving a good fit with experimental data (average RSTotal of 162 and R2 of 0.979) across various xylose concentrations (10–100 g/L) and glucose levels (1–10 g/L). The model was successfully applied to predict xylitol production from non-detoxified corn cob hemicellulosic hydrolysate, showing strong agreement with experimental results (RSTotal of 368 and R2 of 0.988) [50].

Systems biology studies the properties and interactions of organisms using quantitative and qualitative methods, integrating experimental data with mathematical models. It combines omics data (genomics, transcriptomics, proteomics, and metabolomics) to understand cellular and organismal growth, adaptation, and development [51].

In relation to xylitol production, there have been studies that analyze global and specific expression. The transport of xylose by *Candida tropicalis* stands out. Through computational analyses and gene expression studies, 11 candidate xylose transporters were discerned and characterized within the genomic framework of *C. tropicalis,* among which *CtXUT1* exhibited peak expression during the intervals of maximal xylose uptake (21 to 36 h). The results revealed that *CtXUT1* is a key transporter, especially under conditions where xylose is the main carbon source, achieving consumption rates of up to 1.02 g/L/h. This suggests that optimizing xylose transporters could significantly enhance the efficiency of xylitol production [52]. Additionally, studies have shown that pH changes impact the expression levels of this gene, with higher pH values potentially reducing its efficiency. This underscores the critical role of pH control in regulating xylose transport and metabolism in *C. tropicalis* [53].

Genomic and transcriptomic investigations have yielded comprehensive understanding regarding the genetic architecture of *C. intermedia* and its capability to metabolize xylose into commercially significant products such as xylitol. The genomes of two distinct strains, CBS 141442 and PYCC 4715, were subjected to examination, uncovering a notable similarity in size (13.1–13.2 Mbp) and protein-coding gene quantity (5936–6073 genes). Notwithstanding this resemblance, specific genomic alterations unique to each strain, including translocations, were identified, alongside a predominantly conserved chromosomal structure. Furthermore, genomic and transcriptional evaluations elucidated the presence of several xylose reductases, among which is one exhibiting dual specificity for NADH/NADPH cofactors, likely serving a pivotal function in cofactor recycling during the fermentation of xylose [54].

As previously reported, *C. tropicalis* demonstrates significant resistance to inhibitors generated during the hydrolysis of lignocellulosic biomass, where xylose becomes accessible. Initial studies on specific expression confirmed that the alcohol dehydrogenase 1 gene is upregulated in response to furfural, underscoring its critical role in the detoxification process. Following furfural depletion, *ctADH1* transcription was repressed by ethanol to maintain redox balance. Understanding the molecular mechanisms underlying furfural tolerance in *C. tropicalis* is essential for optimizing xylose fermentation processes [55].

Through directed evolution, *C. tropicalis* strains were increased tolerance to inhibitory compounds from lignocellulosic biomass hydrolysis. The *C. tropicalis* Y31-N strain, derived from the parental strain Y-3, showed an 84.8% increase in xylitol production and a fivefold higher furfural consumption rate. This improved performance was attributed to the regulation of cell membrane fluidity through changes in fatty acids, which allowed maintaining the integrity of membrane function under furfural stress. Furthermore, the strain exhibited rapid ATP and NAD(P)H production, supported by a metabolomic analysis that highlighted metabolic rearrangement as a key response [56]. In addition, *C. tropicalis* SHC-03, its evolution toward increased tolerance to inhibitory compounds derived from corn stover hydrolysis was investigated through stepwise evolution. After 60 generations, the evolved strain GB-60 exhibited superior tolerance and reduced subcellular damage against inhibitors such as acetic acid, phenol, and furfural. Comparative transcriptomic and genomic analyses between the GB-60 strain and the parental SHC-03 strain revealed overexpression and mutations in key genes related to cell wall biosynthesis and remodeling, the pentose phosphate pathway, detoxification of inhibitory compounds, and multidrug/multixenobiotic resistance transporters. These results establish a foundation for the development of enhanced strains aimed at optimizing lignocellulosic biomass fermentation [57].

For metabolomics, it was investigated the response of *C. tropicalis* SHC-03 to the toxic byproducts of corn stover hydrolysate. An accumulation of 84.61% reactive oxygen species and mitochondrial damage was observed, inhibiting its growth by 40%. Metabolically, the glutathione and thioredoxin systems were activated, ergosterol biosynthesis and membrane thickness increased, also the expression of transporters and aldehyde reductases was upregulated, contributing to higher inhibitor tolerance [58]. Additionally, through metabolomic analysis in the hydrolysate, 1469 substances were identified, including sugars, aldehydes, acids and phenols that could influence the inhibition of yeast growth. At the cellular level, *C. tropicalis* SHC-03 accumulated reactive oxygen species and maintain endoplasmic reticulum homeostasis. While genes associated with the reduction of glutathione, ergosterol, ubiquinone-n biosynthesis, degradation of misfolded proteins and fatty acids, were found to be upregulated, suggesting that these mechanisms generate a response to detoxify byproducts in the hydrolysate that inhibit cell growth and survival [59].

## Conclusions

Xylitol offers health benefits, including suitability for diabetics, tooth decay prevention, and ear infection reduction. Research on xylitol production focuses on yeast-based processes, particularly *C. tropicalis* and *C. guilliermondii*, as alternatives to chemical methods due to better efficiency and lower environmental impact. Despite high efficiency, industrial-scale production faces challenges like growth inhibition and by-product generation. Overexpression of the XR gene has enhanced xylitol output, suggesting genetic modifications could improve industrial efficiency. Ongoing research and industry-academia collaboration are key to overcoming production challenges.

## Supplementary Information

Below is the link to the electronic supplementary material.

Supplementary file1 (DOCX 12 KB)
