# Supplementary material for: Xylitol: A Promising Sweetener Produced by Candida sp
Source: Indian J Microbiol. 2025 Mar 15;66(4):833–42. doi: 10.1007/s12088-025-01456-1 (PMC13447656; doi:10.1007/s12088-025-01456-1)
Supplement: Supplementary file 1 [file 12088_2025_1456_MOESM1_ESM.docx]

**Table S1** Estimated parameters of kinetic models related to xylitol production in yeasts of the genus *Candida sp*.

| **Parameter** | **Reference** | | | | | | |
| --- | --- | --- | --- | --- | --- | --- | --- |
|  | **[1]** | | **[2]** | | | **[3]** | **[4]** |
| µ_max_ (h^-1^) | 0.104 | NR | 3.259 | 0.255 | 1.691 | NR | 0.12 |
| *K*_s_ (g/L) | NR | NR | 589.1 | NR | NR | NR | NR |
| *B* (g/L) | NR | NR | NR | 1.884 | NR | NR | NR |
| *K*´_s_ (g/L) | NR | NR | NR | NR | 305.9 | NR | NR |
| µ_max_ _glc_(h^-1^) | NR | NR | NR | NR | NR | 0.662 | NR |
| µ_max_ _xit_(h^-1^) | NR | NR | NA | NR | NR | 0.189 | NR |
| *q_max xyl_* (g _xilosa_/g _MSC_*h) | NR | NR | NR | NR | NR | 0.342 | NR |
| *q_max glc_* (g _xilosa_/g _MSC_*h) | NR | NR | NR | NR | NR | 3.276 | NR |
| *K_S, xyl_* (g/L) | NR | NR | NR | NR | NR | 11.761 | NR |
| *K_S, glc_* (g/L) | NR | NR | NR | NR | NR | 9.998 | NR |
| *K_S, xit_* (g/L) | NR | NR | NR | NR | NR | 16.068 | NR |
| *K_i, xyl_* (g/L) | NR | NR | NR | NR | NR | 14.780 | NR |
| *K_i, glc_* (g/L) | NR | NR | NR | NR | NR | 0.100 | NR |
| *K_r_* (g/L) | NR | NR | NR | NR | NR | 0.100 | NR |
| *P_xit_* (m/s) | NR | NR | NR | NR | NR | 7.591X10^-9^ | NR |
| *A* (nil) | NR | NR | NR | NR | NR | NR | 0.266 |
| *δ* (g/L*h) | NR | NR | NR | NR | NR | NR | 0.01 |
| *KO_2_ (g/L)* | NR | NR | NR | NR | NR | NR | 0.002 |
| *Keq (g/L)* | NR | NR | NR | NR | NR | NR | 1.29 |
| *Kin, δ (g/L)* | NR | NR | NR | NR | NR | NR | 0.001 |
| *Kin, S (g/L)* | NR | NR | NR | NR | NR | NR | 393.07 |
| *Kin, P (g/L)* | NR | NR | NR | NR | NR | NR | 376.86 |
| *Kin, O_2_ (g/L)* | NR | NR | NR | NR | NR | NR | 25.15 |
| *Kli, δ (g/L)* | NR | NR | NR | NR | NR | NR | 116.25 |
| *Kli, P (g/L)* | NR | NR | NR | NR | NR | NR | 25 |
| *Kli, S (g/L)* | NR | NR | NR | NR | NR | NR | 50 |
| *Kli, O_2_ (g/L)* | NR | NR | NR | NR | NR | NR | 0.0009 |
| *N_CD_ (rpm)* | NR | NR | NR | NR | NR | NR | 188 |
| *Y_X/S_ (g/g)* | 0.111 | 0.081 | NR | NR | NR | NR | 0.12 |
| *Y_O2_ (g/g)* | 0.657 | 0.507 | NR | NR | NR | NR | 0.51 |
| *α (g/g)* | NR | 7.955 | NR | NR | NR | NR | NR |
| *Y_P/S_ (g/g)* | NR | 0.644 | NR | NR | NR | NR | 0.36 |
| *R^2^* | NR | NR | 0.9946 | 0.9946 | 0.9945 | NR | NR |

NR No information is reported.

[1]	J. S. Aranda-Barradas, M. L. Delia, and J. P. Riba, “Kinetic study and modelling of the xylitol production using *Candida parapsilosis* in oxygen-limited culture conditions,” *Bioprocess Eng.*, 2000.

[2]	W. B. Aguiar, L. F. F. Faria, M. A. P. G. Couto, O. Q. F. Araújo, and J. N. Pereira, “Growth model and prediction of oxygen transfer rate for xylitol production from D-xylose by C. guilliermondii.,” *Biochem Eng J*, vol. 12, pp. 49–59, 2002

[3]	W. Tochampa, S. Sirisansaneeyakul, W. Vanichsriratana, P. Srinophakun, H. H. C. Bakker, and Y. Chisti, “A model of xylitol production by the yeast *Candida mogii*,” *Bioprocess Biosyst. Eng.*, 2005.

[4]	N. L. Mohamad, S. M. Mustapa Kamal, M. N. Mokhtar, S. A. Husain, and N. Abdullah, “Dynamic mathematical modelling of reaction kinetics for xylitol fermentation using *Candida tropicalis*,” *Biochem. Eng. J.*, 2016.
